# Amputation for Traumatic Gangrene of the Foot

**Published:** 1882-08

**Authors:** C. A. Simpson

**Affiliations:** Roswell, Ga.


### AMPUTATION FOR TRAUMATIC GANGRENE OF THE FOOT.

By C. A. SIMPSON, M.D., Roswell, Ga.

On the 24th of last January Dr. ——— sent me a note requesting me to see S—— S——, who, he said, wished me to attend him.

S———, colored, aged 20, laborer, three weeks before had accidentally cut himself with an ax across the instep of the left foot, the wound extending from the anterior edge of the internal to a point an inch in front of the external maleolus, and probably dividing the flexor tendons, anterior tibal artery with accompanying vein, and external saphenous vein. The severe hemorrhage, I am told, was checked with muriated tincture of iron, a compress and bandage applied, and a strap passed around the ball of the foot and fastened at the waist to keep the foot flexed. The details of the treatment until the case came to my care I do not know.

I visited the patient as soon as convenient. I found him very much emaciated, anxious expression, pulse 120. There was a gaping, non-granulating wound, located as described above, discharging a thin unhealthy pus, and a space on the dorsum of the foot extending from the wound forward some two and a half inches by two inches across, free of cuticle; the tissues beneath of a pale, unhealthy sodden appearance, and discharging a thin watery pus. Tactile sensibility was very much diminished all over the dorsum of the foot, while the toes were almost devoid of any feeling.

This was a very unpromising state of affairs. In justice to the physician who first had charge of the case, I must say that the patient could not be kept in bed, but would get up and hobble over the floor on crutches, thus probably tearing open the wound and preventing union.

I enveloped the foot in a thick layer of cotton batting, except the wound and the denuded space on the dorsum, applied a stiff pasteboard splint to keep the foot flexed, and over this a loosely applied plaster of paris bandage. The wound and denuded space were dressed with carbolized linseed oil.

On the 27th, three days after I took charge of the case, bullæ appeared on the dorsum of the foot near the toes. There was no improvement in the appearance of the wound, no indication of an attempt at granulation, and within the next two days the toes became decidedly gangrenous, the tissues becoming soft and breaking down under the probe. The patient was growing more emaciated and weaker every day, with the pulse almost constantly at 120 or beyond.

The foot could not be saved, nor would the condition of the patient warrant waiting for a "line of demarcation," so I determined to operate. With competent assistants and the patient under chloroform, I amputated by the ordinary flap method at the lower third of the leg. This was the nearest point at which I could get anything like sound tissues to make the flaps, but finding the veins occluded with thick, black, clotted blood, and doubtful of the soundness of the tissues, I immediately amputated again at the upper third, making a short anterior and long posterior flap. The blood in the veins here was black, but more fluid than lower down. After ligating the arteries and sponging the flaps with a two and a half per cent. solution of carbolic acid, they were brought together with a sufficient number of interrupted silk sutures, a cloth wet with a solution of carbolic acid was placed over the end of the stump, and a rubber bandage applied.

A very small quantity of blood was lost, and the patient came out well from the chloroform. Anodynes and stimulants were given as required, with milk, eggs, etc., and quinine, grs. xv–xx, per day for several days, till danger of septicæmia had passed. The stump was moistened with carbolic lotion for the first few days, and then ordinary tar water was used. The stump healed rapidly and satisfactorily, most of it by first intention, except a small ulcerated point through the skin made by the sharp edge of the tibia which I neglected to round off.

The patient was reduced so low that others predicted that he would not recover. I was very doubtful myself, but he gradually improved, and now with a wooden leg—the best artificial limb for a laborer—he is putting in full time making mortar for the building of a cotton factory.

I have given as briefly as possible the report of a case which has been of considerable interest to me.

I am satisfied if I had waited for the line of demarcation, as laid down in the books, this patient would have died. A speedy operation was the only thing that could save his life.

If the amputation had not been performed high up, there would have been great danger of sloughing, with the possible necessity of an amputation higher up, and under less favorable conditions.

Sometime after this operation I saw in the February number of the *N. Y. Medical Abstract* an extract from a clinical lecture by Jonathan Hutchison, F.R.C.S., delivered at the London Hospital.

He says: "In cases of traumatic gangrene, ought amputation to be performed without waiting for a line of demarcation? I believe that the reply of most surgeons will be unhesitatingly in the affirmative. Such certainly would be my own.

"The main reason for prompt amputation in such cases is, that the gangrenous process is very dangerous. Whilst the soft parts are dying and the circulation still going on to some extent through them, the blood becomes poisoned by the absorption of gases and fluids from the putrescent parts, and a most dangerous condition of septicæmia results.

"Of this state a rapid pulse, a sunken countenance, high temperature, and vomiting are the most constant signs. It is remarkable how quickly they are sometimes relieved by the removal of the dying part. It may be that the process of mortification is also extended by shock to the nervous system, but I suspect that the chief part of the mischief is done through the blood.

"In the pyæmia which results from phlebitis it is no use to amputate after once the poisonous emboli have been shed from the inflamed vein into the blood. It is then too late, for the secondary abscesses will form, whether you remove the original focus or not.

"In septicæmia from gangrene, however, the the case is different. Here it seems to be possible for the blood to rid itself of the contamination.

"I well remember a young soldier who was under treatment some years ago for a damaged foot, the consequence of a Canadian frost-bite. He had, also, obliteration of his femoral artery. My junior colleague at the time amputated through the tarsus. The stump never healed, and sometime later I amputated in the upper third of the leg at a great distance from the disease, for the whole of his leg looked at the time as healthy as yours or mine. I went high up, because I knew the femoral artery was occluded. The result, however, was, that the stump passed into gangrene, and very soon we had all the symptoms of the most severe form of that malady. The patient had frequent vomiting, a very rapid pulse, and was indeed in such a critical state when (on the third day,) I decided to amputate again, that I did not dare to have him taken from his bed. The second amputation, performed high up in the thigh, saved his life. No ill symptoms occurred after it, and the stump healed well.

"I am inclined to believe that the usefulness of amputation in gangrene will become more widely appreciated, and that this measure will be resorted to, not exclusively in traumatic gangrene, but in all forms attended by serious constitutional symptoms. If a part be simply passing quietly into a mummified condition, and the patient's health not suffering, there is no reason for interfering until you see where nature is going to make the separation. There is indeed no reason for interfering at all, for you must let nature finish the work. If you amputate near the line of demarcation, your stump is almost certain to slough, and all that you must dare do in the way of help in such cases is to just saw through the bones when they are laid bare. The explanation of disappointment in amputating for gangrene, whether traumatic or otherwise, is, I feel sure, almost always from amputating too near the disease."

